# Efficacy and safety of Xianling Gubao capsule in treating postmenopausal osteoporosis: A protocol for systematic review and meta-analysis

**DOI:** 10.1097/MD.0000000000023965

**Published:** 2021-01-08

**Authors:** Fanglian Lou, Siping Xian, ZhongJun Shu, Zhouhai Zheng

**Affiliations:** People's Hospital of Nanchuan District Chongqing, Chongqing, Chongqing, China.

**Keywords:** meta analysis, postmenopausal osteoporosis, protocol, systematic review, xianling gubao

## Abstract

**Background:**

postmenopausal osteoporosis is a systemic metabolic skeletal disease associated with menopause-related estrogen withdrawal. postmenopausal osteoporosis is characterized by low bone mass, bone microstructure destruction, leading to increased bone brittleness and be prone to fracture, resulting in disability and death. At present, the commonly used drugs are estrogen, calcium, bone formation promoter and bone resorption inhibitor, and the side effects are obvious. In Traditional Chinese medicine, kidney-tonifying differentiating medicine is guided by the whole concept, Xianling Gubao capsule as the representative, the treatment of postmenopausal osteoporosis has certain therapeutic advantages, but lacks evidence-based medicine evidence. The purpose of this study is to systematically study the efficacy and safety of Xianling Gubao capsule in the treatment of postmenopausal osteoporosis.

**Methods:**

use computer to search English databases (PubMed, Embase, Web of Science, the Cochrane Library) and Chinese databases (China Knowledge Network, Wanfang, Weipu, Chinese Biomedical Database), in addition manually search Baidu academic, Google academic, from the establishment of database to October 2020, for randomized controlled clinical study of postmenopausal osteoporosis in the Xianling Gubao capsule treatment. Two researchers independently did the data extraction and literature quality evaluation, using RevMan5.3 software to do meta-analysis of the included literature.

**Results:**

this study assessed the efficacy and safety of xianling gubao capsule in the treatment of postmenopausal osteoporosis by total effective rate, bone density after treatment, blood calcium level after treatment, blood phosphorus level after treatment, pain score, quality of life and so on.

**Conclusion:**

this study will provide reliable evidence-based evidence for the clinical application of Xianling Gubao capsule in the treatment of postmenopausal osteoporosis.

**OSF Registration number:**

DOI 10.17605/OSF.IO/TP394

## 1 Introduction

Postmenopausal osteoporosis (PMOP) is a common and frequent age-related metabolic bone disease in perimenopausal women. It is a systemic disease characterized by low bone mass, bone microstructure destruction, leading to increased bone brittleness and be prone to fracture.^[1,2]^ The incidence rate is increasing year by year, and the incidence rate of women over 50 years of age is 30%.^[3]^ Fractures and complications caused by fractures greatly increase the rate of disability and mortality, seriously affecting the quality of life of patients around the world. PMOP has no specific drugs, currently commonly used for estrogen, calcium, bone formation promoters and bone resorption inhibitors and so on,^[4]^ which has certain curative effect, but easy to cause complications of other systems, especially estrogen has great side effects. Under the guidance of “holistic concept” and “syndrome differentiation and treatment ”, Chinese medicine can take care of condition from the whole body, effectively relieve a series of osteoporosis symptoms such as bone pain and weakness, avoid the damage of other functions of the body, and the toxic side effects are small, provide a broad market for its treatment of PMOP. Xianling Gubao Capsule is a traditional Chinese medicine for preventing and treating osteoporosis.^[5,6]^ Taking Xianling Gubao capsule for a long time can improve bone mineral density in postmenopausal osteoporosis patients in varying degrees and the effective rate of treatment of primary osteoporosis is 93.75%.^[7]^

The results of several randomized controlled studies^[8,9]^ show that Xianling Gubao capsule can increase bone mineral density, relieve symptoms, determine curative effect, have less side effects and have abundant drug sources. However, there are differences in the research scheme and curative effect of each clinical trial, which leads to the uneven results, which to some extent affects the promotion of the therapy. Therefore, this study plan to systematically evaluate the efficacy and safety of Xianling Gubao capsule in the treatment of postmenopausal osteoporosis, and provide a reliable reference for the clinical application of Xianling Gubao capsule in the treatment of postmenopausal osteoporosis.

## 2 Methods

### 2.1 Protocol register

This protocol of systematic review and meta-analysis has been drafted under the guidance of the preferred reporting items for systematic reviews and meta-analysis protocols. Moreover, the protocol has been registered on the open science framework (OSF) on November 26, 2020. (Registration number: DOI 10.17605/OSF.IO/TP394).

### 2.2 Ethics

For this protocol, we had no patient recruitment and personal information collection, so the approval of the ethics committee was not required.

### 2.3 2.3Eligibility criteria

#### 2.3.1 Types of studies

Domestic and foreign published randomized controlled trials (RCTs) on Xianling Gubao capsule for postmenopausal osteoporosis. Whether or not blind method was used; language was limited to Chinese and English; publication status was unlimited.

#### 2.3.2 Research subjects

According to the criteria recommended by the WTO, women who were postmenopausal and clearly diagnosed with primary osteoporosis, learned about the study and volunteered. Among them, nationality, race, course of disease, and so on were unlimited.

#### 2.3.3 Interventions

The control group was treated with conventional therapy, and the treatment group was treated with Xianling Gubao capsule on the basis of conventional treatment. Conventional therapies include bisphosphonates, hormones, calcitonin and calcium and vitamin D supplements and so on. There was no limit on the types, dosage, frequency and course of conventional therapy.

#### 2.3.4 Outcome indicators

(1) Primary outcome: ①the overall effective rate, total effective rate = (cure number + effective number)/ total number ∗100%. Efficacy criteria: significant effect: low back pain symptoms disappeared, bone mineral density increased; effective: symptoms of low back pain obviously improved, bone mineral density increased not obvious; invalid: low back pain symptoms and bone mineral density were not improved than before treatment.
(2) Secondary outcomes:bone density after treatment, blood calcium level after treatment, blood phosphorus level after treatment, pain score, quality of life.

### 2.4 Exclusion criteria

(1) Duplicate published studies;
(2) Studies published as abstracts or with incomplete data and unable to obtain complete data after contacting the author;
(3) Literatures of which randomization or allocation assessed as high risk of bias;
(4) Literatures in which intervention combined with other traditional Chinese medicine, Chinese patent medicine, and so on;
(5) No other diseases that interfere with bone metabolism;
(6) Literatures with no related outcome indicators;

### 2.5 Search strategy

“Xianlinggubao,” “ gu zhi shu song ”(osteoporosis),“ jue jing hou gu zhi shu song ”(Postmenopausal osteoporosis),“yuan fa xing gu zhi shu song”(primary osteoporosis),“ lao nian xing gu zhi shu song”(senile osteoporosis) as the Chinese key words, “Xianlinggubao,” “XLGB,” “Osteoporosis,” “Postmenopausal osteoporosis,” “senile osteoporosis,” “primary osteoporosis” as the English key words, use computer to search Chinese databases (China Knowledge Network, Wanfang, Weipu, Chinese Biomedical Database) and English databases (PubMed, Embase, Web of Science, the Cochrane Library), in addition manually search Baidu academic, Google academic, from the establishment of database to October 2020, for all domestic and foreign randomized controlled clinical study of postmenopausal osteoporosis in the Xianling Gubao capsule treatment. For example, PubMed retrieval strategy is shown in Table 1.

**Table 1:** Retrieval strategy of PubMed.

Number	Search terms
1	Xianlinggubao [Title/Abstract]
2	XLGB [Title/Abstract]
3	1 OR 2
4	Osteoporosis [MeSH]
5	Postmenopausal osteoporosis [Title/Abstract]
6	Post-Menopausal Osteoporosis[Title/Abstract]
7	Senile osteoporosis [Title/Abstract]
8	Primary osteoporosis [Title/Abstract]
9	4 OR 5 OR 6 OR 7 OR 8
10	3 AND 9

### 2.6 Data screening and extraction

**Figure 1:**
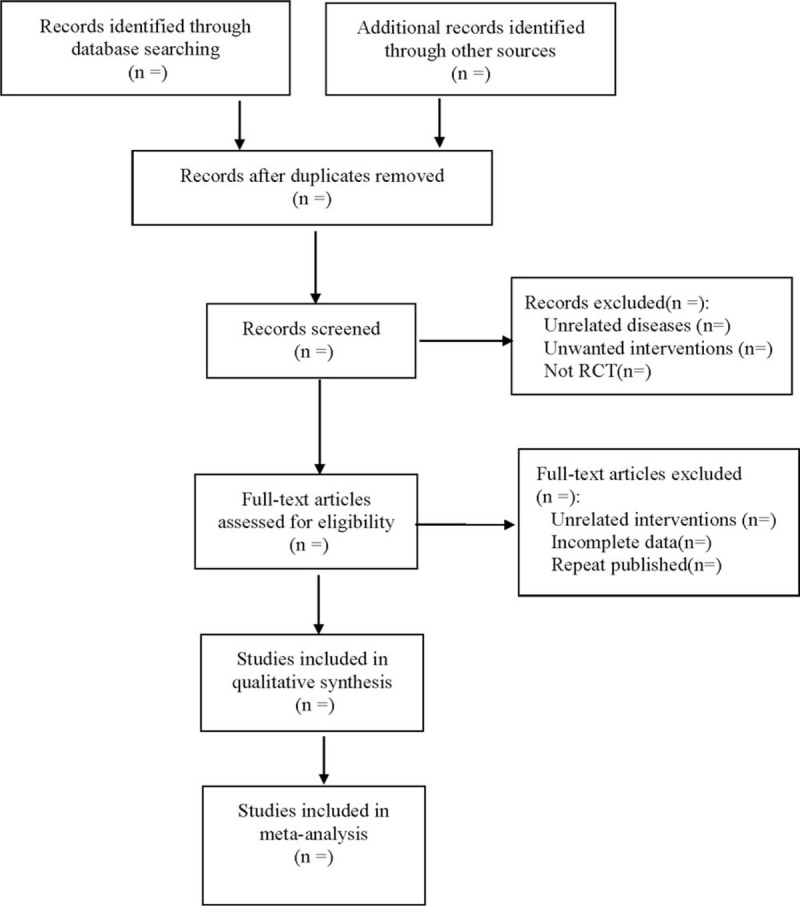
The process of literature screening.

Two researchers used the same self-made form to extract data independently according to inclusion and exclusion criteria, referring to the method of research selection in version 5.0 of the Cochrane collaboration Network system Evaluator Manual, according to the preferred reporting items for systematic reviews and meta-analyses protocols flow chart, and finally cross-checked each other, extracted results together. Inconsistencies were determined through panel discussions. The extracted data include:

(1) General characteristics of clinical research (title, first author, year of publication, sample size);
(2) Information on subjects (mean age, duration of menopause, mean course of disease, stage);
(3) Intervention measures (type, route, dosage, frequency, course of treatment of conventional therapy in treatment and control group; the dosage, route, frequency and course of treatment of Xianling Gubao in the treatment group);
(4) Risk bias assessment factors in randomized controlled trials;
(5) Outcome indicators. The literature screening process is shown in Figure 1.

### 2.7 Literature quality assessment

Evaluation of the quality of the included literature was conducted by using the bias risk assessment tool recommended in Cochrane System Evaluator Manual 5.3, which includes:

(1) random methods;
(2) allocation concealment;
(3) Blindness for researchers and participants;
(4) Blindness of research results;
(5) Integrity of the resulting data;
(6) Selective reporting of research findings;
(7) The existence of other sources of bias.

The results of each evaluation were classified as low bias risk, high bias risk and unclear.

### 2.8 Statistical analysis

#### 2.8.1 Data analysis and processing

Use the RevMan 5.3 software provided by the Cochrane collaboration network to analyze the data. Risk ratio for counting data, mean differences between groups for continuous variable data, both were expressed as 95% confidence interval (95%CI). A χ2 test was used to analyze statistical heterogeneity among studies, when heterogeneity was not statistically significant (*P* > .10, *I*^*2*^ < 50%), the fixed effect model was used; When there was obvious statistical heterogeneity (*P*≤.10, *I*^*2*^ > 50%), the source of heterogeneity was analyzed first, the random effect model was carefully used for combined analysis, and the literature with high bias was deleted by sensitivity analysis, to detect the stability of meta analysis results. After collating, checking and reviewing the data, the Rey Man5. software was used to draw the risk map of bias. The test level of meta analysis was α=0.05.

#### 2.8.2 Dealing with missing data

If there was missing data in the article, contact the author via email for additional information. If the author cannot be contacted, or the author has lost relevant data, descriptive analysis would be conducted instead of meta-analysis.

#### 2.8.3 Subgroup analysis

Subgroup analysis was carried out according to the course of disease; subgroup analysis was carried out according to the course of treatment; subgroup analysis was carried out according to the different conventional therapy drugs in the control group.

#### 2.8.4 Sensitivity analysis

In order to test the stability of meta-analysis results of indicators, a 1-by-1 elimination method will be adopted for sensitivity analysis.

#### 2.8.5 Assessment of reporting biases

The total effective rate, bone density after treatment, serum calcium after treatment and serum phosphorus after treatment were taken as the indexes, and the inverted funnel map was drawn with the standard error of effect quantity as the vertical coordinate. If the inverted funnel diagram was basically symmetrical, it was suggested that there is a small sample effect or publication bias in this study.

#### 2.8.6 Evidence quality evaluation

We choosed to assess the quality of evidence by using the Grading of Recommendations Assessment, Development, and Evaluation, which contains 5 domains, including bias risk, consistency, directness, precision, and publication bias. We would rate the quality of evidence as high, moderate, low, and very low.

## 3 Discussion

Postmenopausal osteoporosis belongs to degenerative osteoporosis, which is caused by increased calcium absorption and decreased bone mass due to the decrease of estrogen, and is also affected by living habits, family heredity, diet structure and so on.^[10–13]^ According to traditional Chinese medicine, osteoporosis belongs to the category of “gu wei ”(bone wilt), which is closely related to kidney, and peri-menopausal women have the characteristics of“ kidney deficiency ”physiologically, so kidney deficiency is considered as the key pathogenesis. Kidney deficiency affects the growth, aging and reproduction of the human body, manifested as endocrine, nerve, immune, metabolic disorders. Long illness can cause qi stagnation and blood stasis, resulting in pain. Therefore, the main treatment of Traditional Chinese medicine is kidney tonifying, accompanied by promoting blood circulation and removing stasis, has the advantages of multi-target,^[14]^ good comprehensive efficacy and small toxic and side effects in the treatment of postmenopausal osteoporosis. Xianling Gubao Capsule, a Chinese patent medicine approved by the State Food and Drug Administration, has been widely used in the prevention and treatment of osteoporosis. Its main components are Yinyanghuo(*Herba Epimedii*), Xuduan(*Radix Dipsaci*), Buguzhi(*Fructus Psoraleae*), Danshen(*Radix Salviae Miltiorrhiae*), Zhimu(*Rhizoma Anemarrhenae*), Dihuang(*Radix Rehmanniae*), among which the Yinyanghuo(*Herba Epimedii*), Xuduan(*Radix Dipsaci*), Buguzhi(*Fructus Psoraleae*) invigorate the kidney, strengthen the muscles and strengthen the bones, Danshen(*Radix Salviae Miltiorrhiae*) activate blood circulation, remove blood stasis, and stop pain, Zhimu(*Rhizoma Anemarrhenae*) and Dihuang(*Radix Rehmanniae*) cool the blood, enrich the blood, and nourish kidney yin, has the effect of nourishing liver and kidney, activating blood circulation and clearing collaterals, strengthening tendons and strengthening bones, has good overall security.^[15]^ Modern pharmacological studies have shown that Yinyanghuo(*Herba Epimedii*) can inhibit osteoclast formation, promote osteoblast proliferation and differentiation, and prevent bone resorption caused by excessive activation of osteoclasts due to abnormal osteoprotegerin/RANKL expression.^[16–18]^ Xuduan(*Radix Dipsaci*) can improve the biomechanical properties of osteoporotic fracture healing callus and promote fracture healing. Dipsacus saponin, Dipsacus decoction or Dipsacus serum can effectively promote the differentiation and proliferation of osteoblasts and prevent the apoptosis of osteoblasts.^[19,20]^ Bakuchiol in Buguzhi(*Fructus Psoraleae*) can also promote the proliferation or differentiation of osteoblasts, increase bone mineral density and promote normal bone development calcification in osteoporosis.^[21]^ Modern studies have found that xianlinggubao mediated anti-osteoporosis effects are mainly related to reactive oxygen species, organic nitrogen reactions and cell migration,^[22]^ which can significantly increase the bone mineral density and serum osteoprotegerin levels of postmenopausal osteoporosis, and then reduce the activation and differentiation of osteoclasts and enhance the degree of apoptosis^[23]^ when applied to postmenopausal osteoporosis.

It is proved that Xianling Gubao capsule is effective in treating postmenopausal osteoporosis. However, the evidence from the RCTs is not consistent, and with the increasing number of clinical trials, it is urgent to systematically evaluate the treatment of postmenopausal osteoporosis. In this study, we will summarize the latest evidence of the efficacy of Xianling Gubao capsule in the treatment of postmenopausal osteoporosis. This work also provides useful evidence for determining the efficacy and safety of Xianling Gubao capsule in postmenopausal osteoporosis patients, which is beneficial to clinical practice and health related decision makers. However, this systematic review has some limitations. There may be some clinical heterogeneity in the different dosage of Xianling Gubao capsule and the varying conventional therapies in the included study. The course of disease is also different, may have a certain impact on the results. Due to the limitation of language ability, we only search English and Chinese literature, and may ignore the research or report of other languages. The sample size is small, mostly in Chinese literature, the quality of the literature is low, most studies do not use blind method correctly, and so on. Conclusions need to be confirmed by large sample, multicenter, high quality RCTs.

## Author contributions

**Data curation:** Fanglian Lou, ZhongJun Shu.

**Funding acquisition:** Zhouhai Zheng.

**Literature retrieval**: Siping Xian and ZhongJun Shu.

**Software:** Siping Xian.

**Supervision:** ZhongJun Shu.

**Writing – original draft:** Fanglian Lou, Siping Xian.

**Writing – review & editing:** Fanglian Lou, Zhouhai Zheng.
